# Engineered NK92 cell-derived exosomes inhibit ovarian cancer progression by degrading *GPRC5A*

**DOI:** 10.3389/fimmu.2025.1613178

**Published:** 2025-11-11

**Authors:** Chaohua Si, Yihan Wang, Yuanyuan Li, Yuqi Chen, Yuxuan Fan, Yunwen Wang, Yanan Tian, Jianen Gao, Xu Ma

**Affiliations:** 1 National Research Institute for Family Planning, Beijing, China; 2 National Human Genetic Resources Center, Beijing, China; 3 Chinese Academy of Medical Sciences & Peking Union Medical College, Beijing, China

**Keywords:** natural killer 92 cells, tLyP-1, exosome, miRNA, siRNA

## Abstract

**Background:**

Natural killer (NK) 92 (NK92) cells are critical immune-effectors with established roles in treating metastatic and hematological malignancies. Owing to the substantial adverse effects, including cytokine release syndrome, associated with NK92 cell therapy, research interest has pivoted toward the safer and potentially more efficient exosome-based approaches. However, the composition, properties, and functions of NK92 cell-derived exosomes remain largely unknown.

**Methods:**

In this study, NK92 cell-derived exosomes were isolated via ultracentrifugation. Small RNA sequencing and proteomic sequencing were performed on both the cells and their exosomes. To enhance exosome targeting to tumor cells, the tLyP-1 targeting peptide was displayed on NK92 cell surfaces through genetic engineering. The mechanism underlying tumor therapy mediated by NK92 cell-derived exosomes was investigated through *in vitro* and *in vivo* experiments. Additionally, we designed a cholesterol-modified ABCB1 siRNA that adsorbs onto exosome surfaces and enters recipient cells to silence target genes.

**Results:**

First, small RNA sequencing and proteomic analysis of NK92 cells and NK92 cell-derived exosomes revealed that the exosomes retained the anti-tumor activity of parental NK cells, inhibiting tumor progression by modulating apoptosis, proliferation, and metastasis. Second, tLyP-1-modified exosomes exhibited enhanced tumor-targeting specificity and exerted anti-tumor effects via the miR-31-5p-*GPRC5A* axis. Furthermore, NK92 cell-derived exosomes effectively delivered ABCB1 siRNA into recipient cells, mediating efficient gene silencing to sensitize chemoresistant ovarian cancer cells to therapeutic agents.

**Conclusion:**

Overall, this study provides a novel strategy to treat ovarian cancer through the preparation of genetically modified NK92 cell-derived exosomes loaded with RNA interference.

## 1 Introduction

Ovarian cancer ranks among the most prevalent gynecologic malignancies globally and represents the second leading cause of gynecologic cancer mortality in women (1, 2). Standard management of ovarian cancer involves surgical resection followed by adjuvant platinum-based chemotherapy (3–5). The efficacy of drug therapy is influenced by multiple factors, including the development of drug resistance in cancer cells. This resistance represents a primary obstacle in ovarian cancer treatment and a key contributor to adverse clinical outcomes (6). The National Comprehensive Cancer Network guidelines (version 1.2023) recommend multiple therapeutic options for drug-resistant ovarian cancer, encompassing several novel agents. As the repertoire of anticancer drugs expands, overcoming resistance by targeting its underlying mechanisms has emerged as a key therapeutic strategy for drug-resistant ovarian cancer; excessive drug efflux represents a major mechanism of such resistance (7, 8). Therefore, targeting ABCB1 to overcome drug resistance in ovarian cancer represents a promising strategy. However, effectively delivering agents to tumors remains a challenging task.

Research has elucidated the application of immune system components, and immune-derived therapeutics have demonstrated an important role in cancer therapy, paving the way for innovative treatment approaches. Exosomes are nanoscale extracellular vesicles (30–200 nm) secreted by diverse cell types that exert biological effects at a distance via the bloodstream (9, 10). Characterized by low immunogenicity, high penetrability, and biocompatibility, these natural carriers function as a delivery vector for cancer therapeutics (11, 12). For example, in a study of breast cancer brain metastases, Kang et al. employed exosomes to co-deliver cPLA2 siRNA and metformin to intracranial glioblastomas, inhibiting tumor cell energy metabolism and growth for cancer therapy (13). In terms of clinical research, Chang et al. conducted human experiments using umbilical cord stem cell exosomes where, after 7 days of aerosol treatment among 24 patients, lung function (forced vital capacity, maximal voluntary ventilation) was significantly improved, and computed tomography scans showed that fibrotic lesions had subsided. The safety was excellent (no serious adverse events), confirming that aerosolized exosomes can reverse the fibrotic process (14). At the same time, the U.S. Food and Drug Administration has approved an investigational new drug application for Purified Exosome Product™ (Rion, Rochester, MN, USA); a phase 1 clinical trial initiated by researchers is currently underway to evaluate its safety as a subcutaneous injection in surgical procedures (NCT0642903). These various investigative efforts suggest that exosomes are in a critical transition period from technological exploration to clinical validation and can be expected to reshape the treatment landscape of tumors and regenerative medicine in the future.

Natural killer (NK) cells represent a promising therapeutic approach in tumor immunotherapy, with NK cells and the exosomes derived from them having been extensively investigated in preclinical and clinical settings to date (15, 16). NK92 cells are central to anti-tumor immune responses. Exosomes derived from these cells retain key anti-tumor properties, such as perforin and granzyme, and offer additional advantages for cancer therapy (17). Notably, NK92 cell-derived exosomes, which are safer than NK92 cells and capable of crossing the blood-brain barrier, achieve extensive biodistribution. Studies demonstrate that exosome engineering can enhance therapeutic payload delivery for disease treatment (18, 19). Consequently, targeted engineering of NK92 cell-derived exosomes may offer a promising strategy for tumor therapy.

This study engineered NK92 cells via gene editing to yield exosomes that target ovarian cancer cells, establishing a cell-free biotherapeutic strategy and advancing the development of NK92-derived exosome-based anticancer therapies.

## 2 Methods

### 2.1 Cell culture and chemicals

For study purposes, all cell lines, including NK92, SKOV3, A2780, and SKOV3/diaminodichloroplatinum (DDP), were obtained from Procell (Wuhan, China) and cultured at 37°C in a humidified atmosphere containing 5% CO_2_. NK92 cells and their derivatives were maintained in X-VIVO medium (Lonza, Basel, Switzerland) supplemented with 10% human platelet lysate and 300 IU/mL of recombinant human interleukin-2. SKOV3 and SKOV3/DDP cells were cultured in McCoy’s 5A medium (Sigma-Aldrich Chemie GmbH, Steinheim, Germany) containing 10% fetal bovine serum. A2780 cells were cultured in RPMI 1640 medium (Gibco, Carlsbad, CA, USA) supplemented with 10% fetal bovine serum (Gibco).

### 2.2 Construction of stable transfer cell lines

The lentiviral vector, designed and synthesized by Genepharm (Shanghai, China), was transfected into NK92 cells following the manufacturer’s lentiviral-transduction protocol. Stable cell lines were subsequently selected using puromycin (2 μg/mL).

### 2.3 Exosome isolation, characterization, and labeling

Cells were incubated in conditioned medium for 48 h at 37°C. The conditioned medium was centrifuged at 500 ×g for 10 min at 4°C, followed by centrifugation at 16, 800 ×g for 30 min at 4°C. The supernatant was filtered through a 0.22-μm filter (Millipore, Burlington, MA, USA) and centrifuged at 120, 000 ×g for 70 min at 4°C. Pelleted exosomes were washed and resuspended in phosphate-buffered saline (PBS). Exosomes were characterized using nanoparticle-tracking analysis (NTA) with a Nicomp Z3000 system (Particle Sizing Systems, Santa Barbara, CA, USA). Morphology was assessed by transmission electron microscopy (TEM) using a JEM-1230 system (JEOL, Tokyo, Japan). For labeling, exosomes were incubated with PKH67 dye (Sigma-Aldrich, St. Louis, MO, USA) according to the manufacturer’s instructions.

### 2.4 Proteomic analysis

NK92 cells and culture supernatants were collected for proteomic sequencing, with three samples per group. Samples were lysed in SDT buffer (4% SDS, 100 mM of Tris-HCl, pH 7.6), sonicated, and heated at 95°C for 15 min. After centrifugation (14, 000 ×g, 15 min), supernatants were quantified using a bovine serum albumin protein assay kit (P0012; Beyotime Biotechnology, Shanghai, China) and stored at −80°C.

For sodium dodecyl sulfate-polyacrylamide gel electrophoresis (SDS-PAGE), 20 μg of protein per sample was mixed with 6× loading buffer, heated at 95°C for 5 min, and separated on 12% polyacrylamide gels. Proteins were visualized using Coomassie Blue R-250 staining.

For mass spectrometry, 100 μg of protein per sample was reduced with 100 mM of DTT (95°C, 5 min). Detergents and low-molecular-weight components were removed via repeated ultrafiltration (Sartorius, 30 kDa, VN01H22) using Universal Assay Buffer buffer (8 M of urea, 150 mM of Tris-HCl, pH 8.5). Reduced cysteine residues were alkylated with 100 mM of iodoacetamide (100 μL in UA buffer; 30 min, dark). Filters were washed with UA buffer (3 × 100 μL) followed by 50 mM of NH_4_HCO_3_ (2 × 100 μL). Proteins were digested with 4 μg of trypsin (Promega Corp., Madison, WI, USA) in 40 μL of 50-mM NH_4_HCO_3_ (37°C, overnight). Peptides were collected as filtrate and desalted using C18 columns. Peptide concentration was determined by ultraviolet absorbance at 280 nm (extinction coefficient: 1.1 for 0.1% g/L of solution), accounting for tryptophan/tyrosine frequency in vertebrate proteins.

Samples were analyzed on a timsTOF Pro mass spectrometer (Bruker, Billerica, MA, USA) coupled to a nanoElute system (Bruker) via a CaptiveSpray source. Peptides were separated on a 25-cm × 75-μm analytical column (1.6-μm C18 beads; IonOpticks, Victoria, Australia) at 50 °C (column oven; Sonation GmbH, Biberach, Germany). After equilibration with four column volumes of buffer A (99.9% H_2_O, 0.1% formic acid; 800 bars), separation was achieved at 300 Nl/min using a linear gradient. MS parameters were as follows: PASEF mode; *m/z*, 100-1700; 1/*K*_0_, 0.75-1.4 V·s/cm² (100-ms ramp); capillary voltage, 1, 500 V; dry gas, 3 l/min (180 °C); 10 MS/MS scans/cycle (1.16 s); charge states, 0-5; active exclusion, 0.5 min; and collision energy, 20–59 eV.

Data were processed in MaxQuant (v1.6.17.0; Max Planck Institute of Biochemistry, Munich, Germany) against a reference database. Notable parameters include: precursor mass tolerance, 6 ppm; fragment ion tolerance, 20 ppm; enzyme, trypsin/P (maximum of two missed cleavages); fixed modification, carbamidomethylation; and variable modifications, N-terminal acetylation and methionine oxidation. Peptide and protein false-discovery rates were set to 1%. Label-free quantification determined protein abundance. Differentially expressed proteins were defined as those exhibiting a fold change of >2 or <0.5 (*P* < 0.05, Student’s *t* test).

### 2.5 Small RNA sequencing

Total RNA from cells and exosomes was isolated using TRIzol reagent (Invitrogen, Carlsbad, CA, USA) according to the manufacturer’s protocol, with a sample size of three per group. Purified total RNA served as the starting material for library preparation. Briefly, 3' and 5' RNA adapters were ligated to respective ends of small RNAs. First-strand cDNA was synthesized using a reverse transcription primer. Double-stranded cDNA libraries were generated by polymerase chain reaction (PCR) amplification; then, purification and size selection were completed to retain inserts of 18–40 bp.

Library quality was assessed by quantifying insert fragments and determining effective library concentrations. Qualified libraries were pooled in equimolar ratios based on effective concentration and sequenced on an Illumina (San Diego, CA, USA) platform to meet target data requirements.

Raw sequencing data were generated by base-calling Illumina fluorescence images, yielding short reads in FASTQ format (Cock et al., 2010), which encode nucleotide sequences and corresponding quality scores. Raw read quality was evaluated using Fastp (v0.23.1; Chen et al., 2018).

Statistically analyze the expression levels of known and novel miRNAs in each sample, and normalize the expression levels using TPM (Zhou et al., 2010). TPM= (read count * 1, 000, 000)/libsize (libsize: total miRNA read count). For samples with biological replicates, use DESeq2 (Love MI et al., 2014) for differential expression analysis between two comparison groups. DESeq2 provides statistical routines to determine differential expression in digital gene expression data using a model based on the negative binomial distribution. For samples without biological replicates, use the edgeR (Robinson MD et al., 2010) TMM algorithm to normalize read count data for analysis.

### 2.6 RNA Sequencing

Total RNA was isolated from cells and exosomes using TRIzol reagent (Invitrogen) according to the manufacturer’s protocol, with a sample size of three per group. RNA concentration and purity were quantified using a NanoDrop ND-1000 spectrophotometer (NanoDrop Technologies, Wilmington, DE, USA), and integrity was assessed with an Agilent 2100 bioanalyzer (Agilent Technologies, Santa Clara, CA, USA). Samples with an RNA integrity number of <7.0 were excluded. Approximately 5 μg of total RNA underwent ribosomal RNA depletion using the Ribo-Zero rRNA Removal Kit (Illumina). Depleted RNA was fragmented via divalent cations at elevated temperatures. Fragmented RNA was reverse-transcribed into first-strand cDNA; second-strand cDNA synthesis was then performed using *Escherichia coli* DNA polymerase I, RNase H, and dUTP (yielding U-labelled second-strand DNA). Double-stranded cDNA fragments underwent end-repair and 3′ adenylation, followed by ligation to indexed adapters. Adapter-ligated fragments were size-selected using AMPure XP beads (Beckman Coulter, Brea, CA, USA). After USER enzyme (NEB, Ipswich, MA, USA) treatment, ligated products were PCR-amplified (95°C for 3 min; 8 cycles of 98°C for 15 s, 60°C for 15 s, 72°C for 30 s; 72°C for 5 min). The resulting cDNA libraries exhibited an average insert size of 300 ± 50 bp. Finally, paired-end sequencing (2 × 150 bp) was conducted on an Illumina HiSeq 4000 platform, following the manufacturer’s guidelines.

Raw data (raw reads) of fastq format were firstly processed through in-house perl scripts. In this step, clean data (clean reads) were obtained by removing reads containing adapter, reads containing ploy-N and low quality reads from raw data. At the same time, Q20, Q30 and GC content the clean data were calculated. All the downstream analyses were based on the clean data with high quality.

Differential expression analysis of two conditions/groups (three biological replicates per condition) was performed using the DESeq2 R package (1.16.1). DESeq2 provide statistical routines for determining differential expression in digital gene expression data using a model based on the negative binomial distribution. The resulting P-values were adjusted using the Benjamini and Hochberg’s approach for controlling the false discovery rate. Genes with an adjusted P-value.

### 2.7 Western blot analysis

Total protein from cell lines or exosomes was extracted using radioimmunoprecipitation assay buffer supplemented with PMSF (Solarbio, Beijing, China) and quantified with a bovine serum albumin kit (Beyotime Technologies). Equal amounts of protein were separated by SDS-PAGE and transferred to polyvinylidene fluoride membranes (Millipore, Burlington, MA, USA). After blocking with 5% bovine serum albumin in TBST, membranes were incubated overnight at 4°C with primary antibodies, followed by 1-h incubation with horseradish peroxidase (HRP)-conjugated secondary antibodies at 37°C. Protein bands were visualized using a chemiluminescence kit (Millipore) and quantified by densitometry.

Primary antibodies included granzyme (13588-1-AP, rabbit polyclonal; Proteintech, Chicago, IL, USA), perforin (14580-1-AP, rabbit polyclonal; Proteintech), CD9 (60232-1-Ig, mouse monoclonal; Proteintech), CD81 (66866-1-Ig, mouse monoclonal; Proteintech), TSG101 (28283-1-AP, rabbit polyclonal; Proteintech), calnexin (10427-2-AP, rabbit polyclonal; Proteintech), and Lamp2b (ab18529, rabbit polyclonal; Abcam, Cambridge, UK).

Secondary antibodies included goat anti-rabbit IgG-HRP (ab6728; Abcam) and goat anti-mouse IgG-HRP (ab205719; Abcam, Cambridge, UK).

### 2.8 Quantitative real-time polymerase chain reaction

Total RNA was extracted with RNAiso Plus (TaKaRa Bio, Shiga, Japan), following the manufacturer’s protocol. RNA integrity and purity were assessed using a NanoDrop One spectrophotometer (NanoDrop Technologies). Reverse transcription was performed using the PrimeScript RT Reagent Kit with gDNA Eraser (TaKaRa Bio). Genomic DNA was eliminated by incubation at 42°C for 2 min. Tissue RNA was reverse-transcribed under the following conditions: 37°C for 15 min and 85°C for 5 s. cDNA was stored at −80°C immediately after synthesis. qRT-PCR was conducted on a QuantStudio 5 real-time PCR system (Applied Biosystems, Foster City, CA, USA) using Hieff qPCR SYBR Green Master Mix (Yeasen Biotechnology, Shanghai, China). Reaction conditions included the following: initial denaturation at 95°C for 5 min; 40 cycles of 95°C for 10 s, and annealing/extension at 60°C for 30 s (with primer-specific annealing temperatures where required). Relative RNA quantification was calculated via the 2 −ΔΔCt method, using GAPDH as the internal reference gene. The primer sequences used were as follows (F, forward; R, reverse): Lamp2b -F: GAAAATGCCACTTGCCTTTATGC; Lamp2b -R: AGGAAAAGCCAGGTCCGAAC; GAPDH-F: AACGGATTTGGTCGTATTGG; GAPDH-R: TTGATTTTGGAGGGATCTCG; ABCB1-F: TTGCTGCTTACATTCAGGTTTCA; ABCB1-R: AGCCTATCTCCTGTCGCATTA; and miR-31-5p-F: AGGCAAGATGCTGGCATAGCT.

### 2.9 Luciferase activity assays

Based on the predicted miR-31-5p binding site, we amplified the 3′UTR sequences of wild-type GPRC5A (GPRC5A-wt) and a site-directed mutant (GPRC5A-mut), then cloned these into the dual-luciferase reporter vector psiCHECK2 (GeneChem, Shanghai, China). HEK293T cells, selected for their high transfection efficiency, were used for all assays. Cells were co-transfected with either the GPRC5A-wt or GPRC5A-mut reporter plasmid, along with miR-31-5p mimic or negative control mimic, using Lipofectamine 3000 (Thermo Fisher Scientific, Waltham, MA, USA). At 48 h post-transfection, cells were harvested, and luciferase activity was quantified using a dual-luciferase reporter assay kit (Beyotime Biotechnology). Firefly luciferase activity served as the internal control for normalizing Renilla luciferase activity.

### 2.10 Preparation of exo^SiABCB1^

Following a 48-h incubation period, cell culture supernatant was collected and exosomes were isolated by ultracentrifugation. The exosome pellet was washed in PBS via ultracentrifugation and resuspended. All centrifugation steps were performed at 4°C. A 30-μg aliquot of exosomes was incubated with 100 μmol of Cy3-labeled cholesterol-modified ABCB1 siRNA in 100 μL of solution for 3 h at 37°C. Unbound siRNA was subsequently removed by washing the exosomes with PBS via ultracentrifugation. The pellet was resuspended in PBS, and fluorescence was measured to quantify siRNA loading. Loading efficiency (%) was determined by comparing sample fluorescence intensity (measured at Cy3’s excitation/emission maxima: 548 nm/570 nm using a Victor3–1420 Multilabel Plate Reader [PerkinElmer, Waltham, MA, USA]) to a standard curve of free Cy3-labeled ABCB1 siRNA.

### 2.11 Cell apoptosis assay

Cell apoptosis was detected using an annexin V-FITC/propidium iodide apoptosis detection kit (Beyotime). The 1 × 10^5^ cells were harvested and double-stained with FITC and propidium iodide after transfection, followed by analysis on a flow cytometer (FACSCalibur; Becton Dickinson, Frankin Lakes, NJ, USA). The acquired data were analyzed and fitted using the FlowJo™ software (Becton Dickinson).

### 2.12 Cell counting kit-8 assay

For the CCK-8 assay, 1500 treated cells were seeded in 96-well plates. After the cells were cultured for 0, 12, 24, 48, 72 and 96 h, the cells were added to a CCK-8 (Dojindo Laboratories, Kumamoto, Japan) and incubated at 37 °C for 2 h. The OD450 value was measured using a microplate detector.

### 2.13 Transwell migration assay

For cell-migration assays, 1.5 × 10^5^ treated cells were seeded in Transwell chambers with an 8-μm well membrane (Corning, Inc., Corning, NY, USA). After 4 h of culture, the lower-layer cells were fixed, while the upper-layer cells were removed by wiping. Migrating cells were stained with Giemsa (Solarbio). Images of the cells were obtained using light microscopy in four fields.

### 2.14 Animal models

All mouse procedures were approved by the Animal Care and Use Committee of the National Research Institute for Family Planning, National Institutes of Health Commissioners. Four-week-old BALB/c nude mice were purchased from Vital River Laboratories (Beijing, China). Following 1-week acclimatization, mice received intraperitoneal or subcutaneous injections of stabilized SKOV3 or SKOV3/DDP cells (2 × 10_6_ cells in 100 μL of PBS), as required by the experimental design. Animals were randomly assigned to experimental groups using a computer-generated random number sequence after baseline assessments. Group allocation was concealed until interventions commenced. Throughout the study, investigators performing surgeries, administering treatments, and assessing outcomes were blinded to group allocation. Coded labels were used for all samples/data, with the key retained by an independent researcher until final analysis. Body weight and activity levels were monitored regularly. Experiments were terminated if mice exhibited severe lethargy, >20% weight loss, or overt cachexia. The mice were anesthetized by intraperitoneal injection with tribromoethanol at a dose of 240 mg/kg. For intraperitoneal tumor models, bioluminescence was quantified using the IVIS Lumina system (Caliper Life Sciences; Hopkinton, MA, USA) after administration of D-luciferin (150 mg/kg; Yeasen Bio, Shanghai, China). At experimental endpoints, mice were euthanized by CO_2_ inhalation (30% flow rate of chamber volume per minute for CO2). Major organs and tumors were harvested, fixed in paraffin, and processed for hematoxylin and eosin (HE) staining or immunohistochemistry (IHC).

### 2.15 IHC

For IHC, paraffin-embedded sections were heated at 60 °C for 2 h, deparaffinized, and subjected to antigen retrieval. Sections were incubated with the following primary antibodies (Proteintech) at 37 °C for 60 min: anti-HE4 (1:500), anti-Ki67 (1:200), anti-vimentin (1:8000), and anti-ABCB1 (1:500). Subsequently, sections were incubated with secondary antibodies at 37 °C for 15 min, followed by HRP-conjugated streptavidin for 10 min. Chromogenic development was performed using 3, 3′-diaminobenzidine, and cell nuclei were counterstained with hematoxylin. Stained sections were visualized and imaged using an Olympus microscope (Olympus Corp., Tokyo, Japan).

### 2.16 Statistical analysis

Data were analyzed using GraphPad Prism 7.0 (GraphPad Software, San Diego, CA, USA) and expressed as mean ± standard deviation values. Differences between two independent groups were compared using unpaired two-sided Student’s *t* tests. Statistical significance was defined as *P* < 0.05. Figures were assembled using Adobe Illustrator 2020 (Adobe Inc., San Jose, CA, USA).

## 3 Results

### 3.1 Isolation and characterization of NK92 cell-derived exosomes

**Figure 1:**
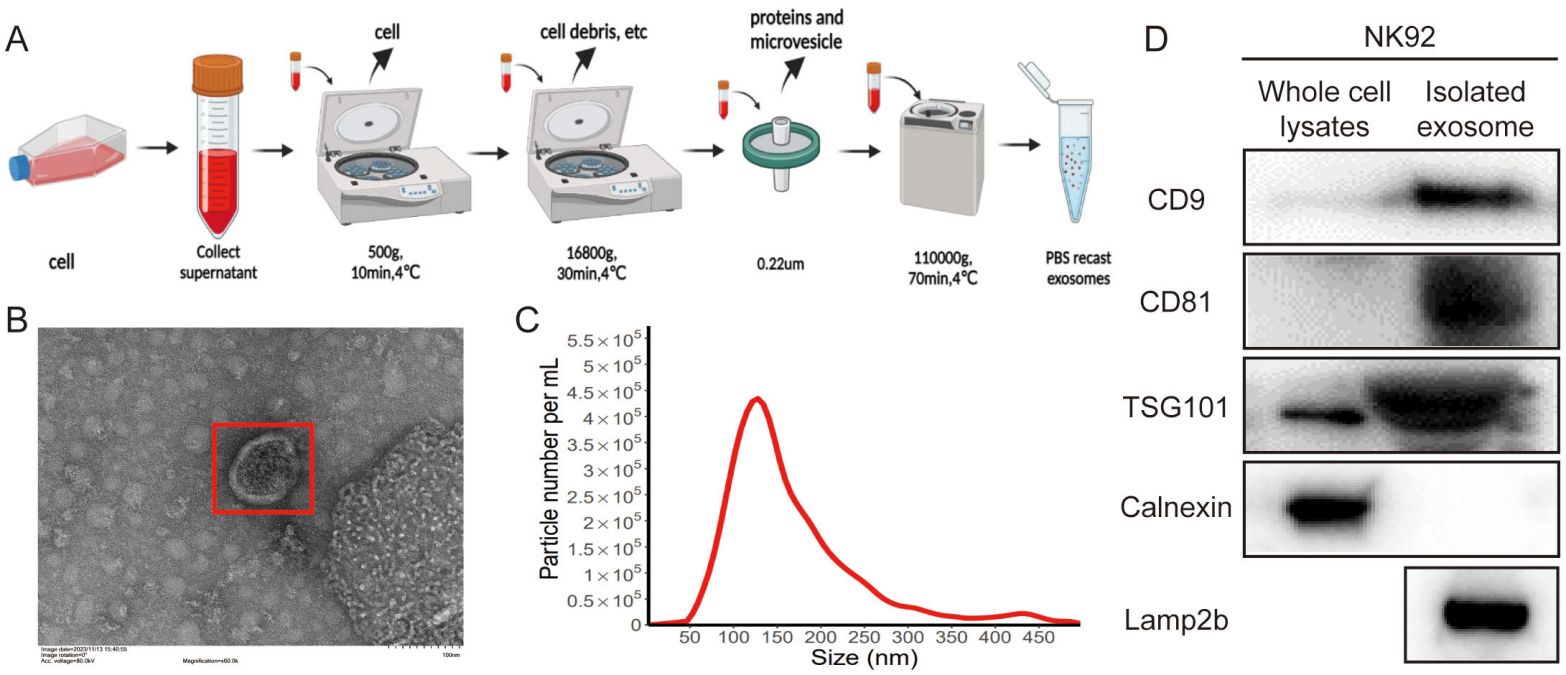
Isolation and characterization of NK92 cell-derived exosomes. **(A)** Schematic of the ultracentrifugation method. **(B)** Representative TEM images of NK92 cell-derived exosomes. Scale bar = 100 nm. **(C)** NTA size distribution analysis of NK92 cell-derived exosomes. **(D)** Western blot of whole-cell lysates and NK92 cell-derived exosomes for CD9, CD81, TSG101, Lamp2b, and the negative control calnexin.

To investigate the role of NK92 cell-derived exosomes in immunotherapy, we isolated and characterized exosomes from NK92 cell supernatants using ultracentrifugation (**Figure 1A**). TEM revealed that the exosomes exhibited a spherical morphology (**Figure 1B**). NTA indicated an average exosome size of 139 nm (**Figure 1C**). Western blot analysis confirmed the presence of exosomal markers (CD9, CD81, TSG101) and the absence of the negative control calnexin in isolated exosomes compared to whole NK92 cell lysates (**Figure 1D**). Furthermore, we detected expression of the lysosomal membrane protein Lamp2b, a potential carrier for targeting peptides in NK92-derived exosomes by western blot analysis (**Figure 1D**).

### 3.2 Proteomic analysis and small RNA sequencing of NK92 cells and derived exosomes

**Figure 2:**
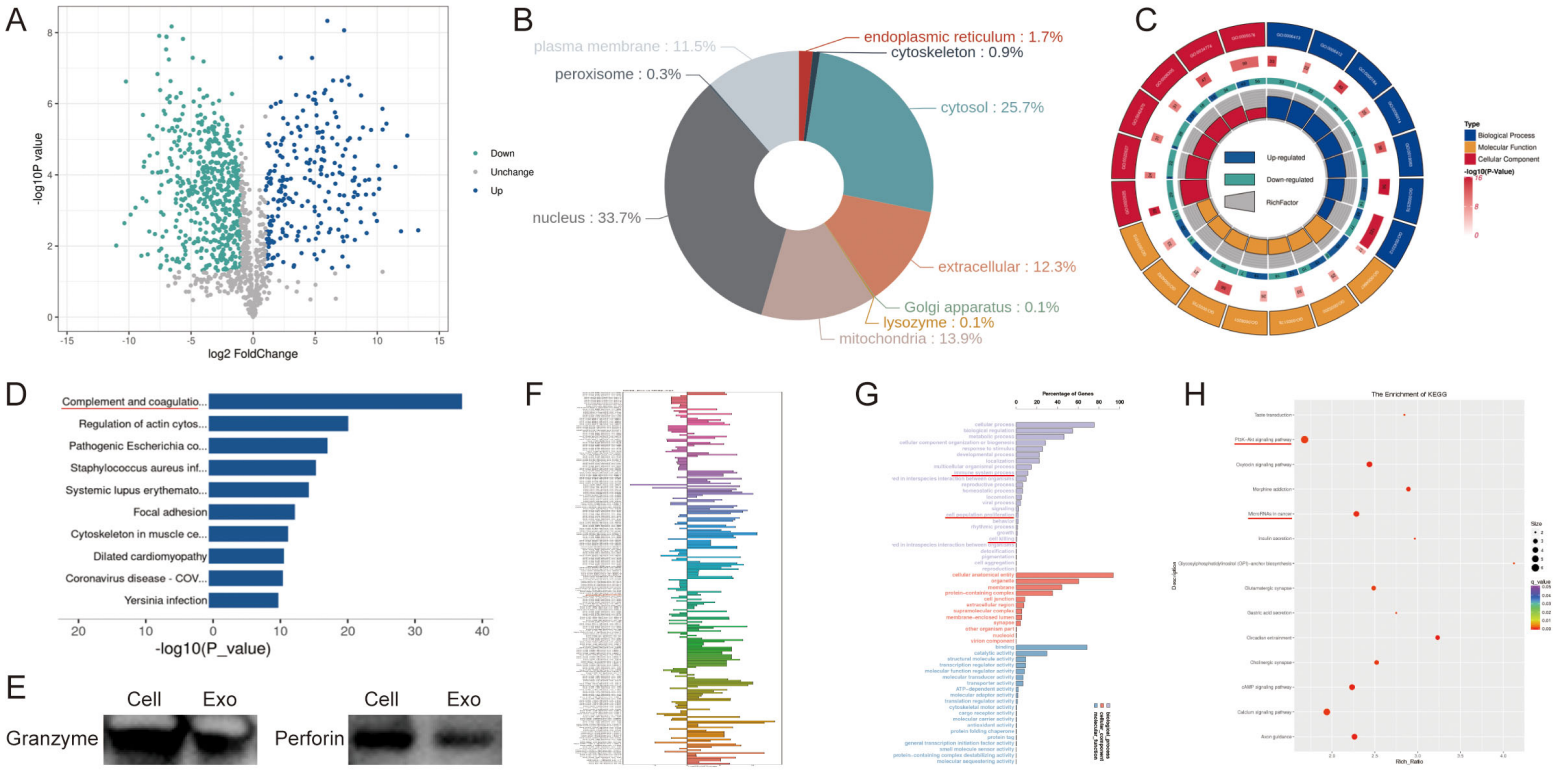
Proteomic analysis and small RNA-seq of NK92 cells and NK92 cell-derived exosomes. **(A)** Volcano plot depicting differentially expressed proteins between NK92 cells (n = 3) and NK92 cell-derived exosomes (n = 3). **(B)** Subcellular localization analysis of differentially expressed proteins in NK92 cells and NK92 cell-derived exosomes. Colors denote distinct subcellular compartments; percentages indicate the proportion of differentially expressed proteins localized to each compartment. **(C)** GO analysis of upregulated differentially expressed proteins in NK92 cell-derived exosomes. Inner circle: Top 20 enriched GO terms color-coded by ontology (biological process, molecular function, cellular component). Second circle: -log_10_(*P* value) for term enrichment. Third circle: Protein ratio per term. Fourth circle: Enrichment factor for each GO term; bar diagrams display exact values. **(D)** KEGG pathway analysis of upregulated proteins in NK92 cell-derived exosomes. Pathways are listed on the vertical axis; the horizontal axis represents enrichment significance (-log_10_[*P* value]). **(E)** Western blot of perforin and granzyme expression in NK92 cells and NK92 cell-derived exosomes. **(F)** Differential miRNA expression profiles in NK92 cells (n = 3) and NK92 cell-derived exosomes (n = 3). **(G)** GO analysis of upregulated miRNAs in NK92 cell-derived exosomes. Vertical axis: Ontology classification (three categories). Horizontal axis: Proportion of target genes annotated to each GO term relative to all annotated targets. **(H)** KEGG enrichment analysis of upregulated miRNAs in NK92 cell-derived exosomes. Point size reflects the number of enriched genes per pathway; color intensity indicates enrichment level (red: highest enrichment).

Current research indicates that NK92 cells mediate tumor cytotoxicity primarily through secreted proteins. To determine whether NK92 cell-derived exosomes retain this functional capacity, we performed comparative proteomic profiling of NK92 cells and their exosomes. We identified 776 differentially abundant proteins, with 231 significantly enriched in exosomes (**Figure 2A**). Subcellular localization analysis revealed 12.3% of differential proteins are secreted extracellular effectors (**Figure 2B**). Gene Ontology (GO) analysis of exosome-enriched proteins demonstrated their predominant involvement in immune system activation and regulation, particularly complement response pathways (**Figure 2C**). Kyoto Encyclopedia of Genes and Genomes (KEGG) pathway analysis further indicated these proteins operate primarily via the complement and coagulation cascades (**Figure 2D**). Notably, we confirmed granzyme and perforin expression within NK92 cell-derived exosomes (**Figure 2E**). Given the established regulatory roles of exosomal RNAs in tumor progression, we characterized miRNA expression profiles using small RNA-seq. Hierarchical clustering revealed distinct miRNA signatures between NK92 cells and their exosomes (**Figure 2F**). We detected 1286 differentially expressed miRNAs, with 885 enriched in exosomes. Upon applying a threshold (|log2FC| ≥ 1.0, *P* < 0.05), 247 miRNAs exhibited significant differential expression (152 upregulated, 95 downregulated; **Figure 2F**). Analysis of the 152 exosome-enriched miRNAs using the dbDEMC database indicated their reduced expression across multiple cancer types (**Supplementary Figure S1A**). This finding, consistent with known functions of non-coding RNAs, suggests that NK92-derived exosomes possess tumor-suppressive potential. Prediction and functional analysis of miRNA target genes revealed that these differentially expressed targets modulate cell proliferation and cytotoxicity through diverse pathways. GO analysis and KEGG pathway analyses substantiated the significant regulatory role of NK92 cells and their exosomes in oncogenesis (**Figures 2G, H**).

### 3.3 Anti-tumor effect of exo^tLyP-1^*in vitro*

**Figure 3:**
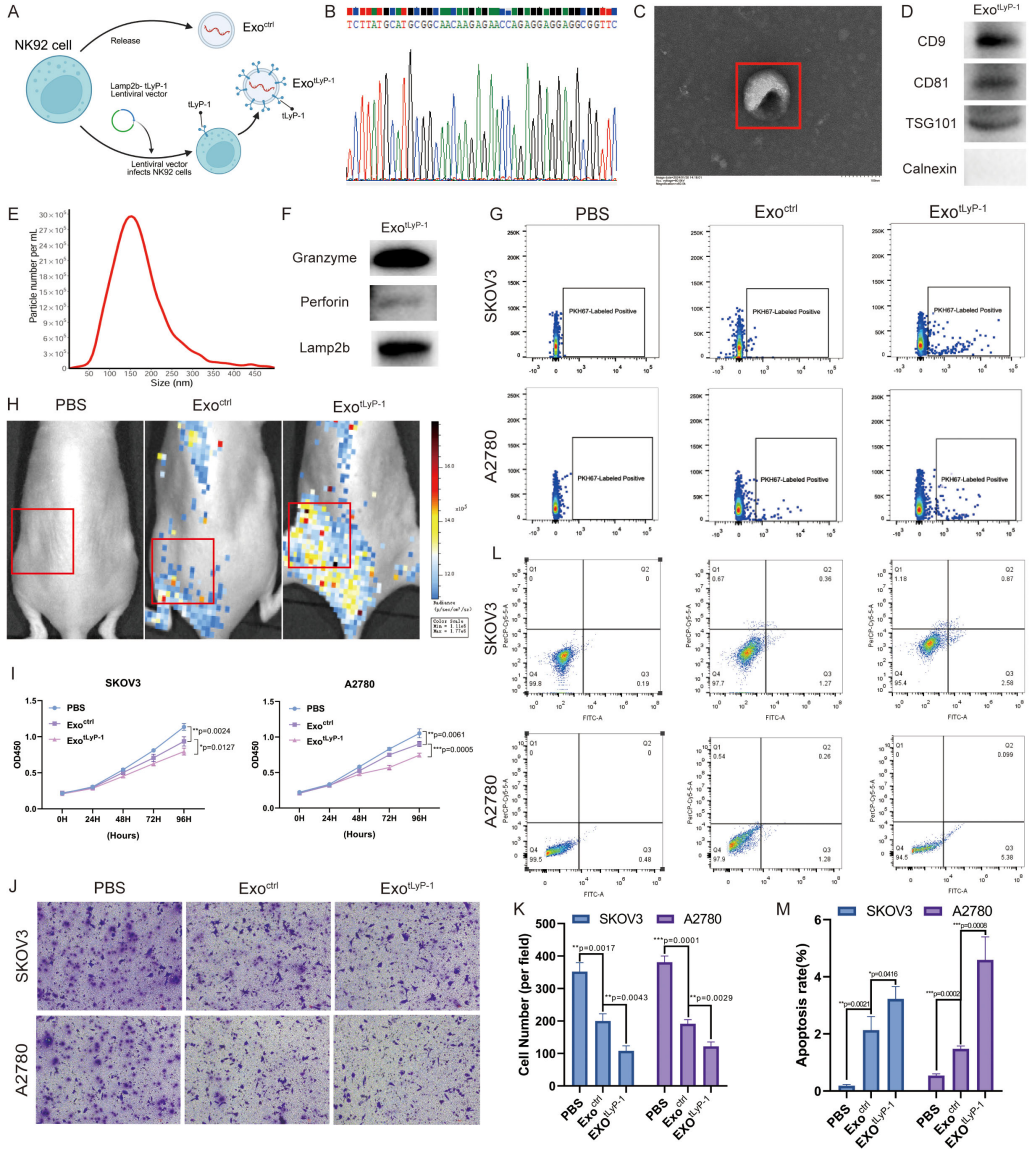
Anti-tumor effect of exo^tLyP-1^*in vitro*. **(A)** Schematic of the construction of engineered NK92 cells. **(B)** Sanger sequencing confirming tLyP-1 modification in NK92 cells. **(C)** TEM images of purified exo^tLyP-1^. Scale bar = 100 nm. **(D)** Western blot of exo^tLyP-1^ for exosomal markers (CD9, CD81, TSG101) and negative control (calnexin). **(E)** NTA size distribution analysis showing the size distribution and concentration of exo^tLyP-1^. **(F)** Western blot of cytotoxic effector proteins (perforin, granzyme) and the membrane surface protein Lamp2b in exo^tLyP-1^. **(G)** Flow cytometry analysis using annexin V-FITC/povidone iodine staining to assess targeting and induction of apoptosis in ovarian cancer cells (1 × 10^4^) *in vitro* following treatment with exo^ctrl^ or exo^tLyP-1^. The PKH67-labeled positive cells shown in the figure represent the positive cells. **(H)***In vivo* fluorescence imaging demonstrating the tumor-targeting capability of exo^ctrl^ (100 μg) and exo^tLyP-1^ (100 μg) in an ovarian cancer xenograft model. Radiance scale: min = 1.11 × 10_6_, max = 1.77 × 10_6_ (p/s/cm²/sr¹). **(I)** CCK-8 assay quantifying the proliferation of ovarian cancer cells treated with exo^ctrl^ or exo^tLyP-1^ (30 μg, n = 4). **(J, K)** Transwell migration assay evaluating the effect of exo^ctrl^ or exot^LyP-1^ (30 μg) on ovarian cancer cell motility (n = 3). Scale bar = 100 μm. **(L, M)** Flow cytometric analysis of annexin V-FITC/povidone iodine staining to determine apoptosis induction in ovarian cancer cells treated with ex^octrl^ or exotLyP-1 (30 μg, n = 3). The total number of cells in regions Q2 and Q3 in the figure represents the total number of apoptotic cells. Data are shown as mean ± standard deviation values; ns *P* ≥ 0.05, *P < 0.05, **P < 0.01, ***P < 0.001, ****P < 0.0001.

Exosomes can be genetically engineered to enhance their tumor-targeting specificity, thereby improving therapeutic efficacy. In this study, we generated NK92 cell-derived exosomes displaying the tLyP-1 targeting peptide (CGNKRTR) on their surface by fusing it with the exosomal membrane protein Lamp2b (**Figure 3A**). Sanger sequencing of genomic DNA extracted from engineered cells confirmed successful integration of the target gene (**Figure 3B**). Engineered NK92 cell-derived exosomes (referred to as exo^tLyP-1^) were isolated via ultracentrifugation. Characterization by TEM, NTA, and western blot confirmed that their physicochemical properties (size, morphology, and presence of exosomal markers) were comparable to those of unmodified exosomes (called exo^ctrl^) (**Figures 3C–E**). Furthermore, the engineered exosomes retained their cytotoxic cargo, including perforin and granzyme (**Figure 3F**).

To assess whether tLyP-1 modification enhanced targeting of ovarian cancer cells, we conducted *in vitro* and *in vivo* experiments. Ultimately, we determined that PKH67-labeled exo^tLyP-1^ exhibited significantly enhanced cellular uptake by tumor cells compared to PBS or exo^ctrl^ (**Figure 3G**). Subsequently, in a subcutaneous tumor mouse model, DIR-labeled exo^tLyP-1^ was systemically administered via tail vein injection. *In vivo* imaging performed 24 h post-injection revealed significantly greater accumulations of exo^tLyP-1^ at the tumor site compared to control mice (**Figure 3H**). These results demonstrate that tLyP-1 modification confers superior tumor-targeting capability.

We next evaluated the functional consequences of exo^tLyP-1^ uptake on ovarian cancer cells. CCK-8 and Transwell assays indicated that co-incubation with exo^tLyP-1^ significantly reduced tumor cell proliferation and migration (**Figures 3I–K**). Apoptosis assays further demonstrated that exo^tLyP-1^ significantly promoted tumor cell death compared to PBS or exo^ctrl^ treatments (**Figures 3L, M**). In summary, exo^ctrl^ exhibit enhanced tumor-targeting specificity and showed potent anti-tumor efficacy against ovarian cancer cells.

### 3.4 *In vivo* anti-tumor effect of exo^tLyP-1^

**Figure 4:**
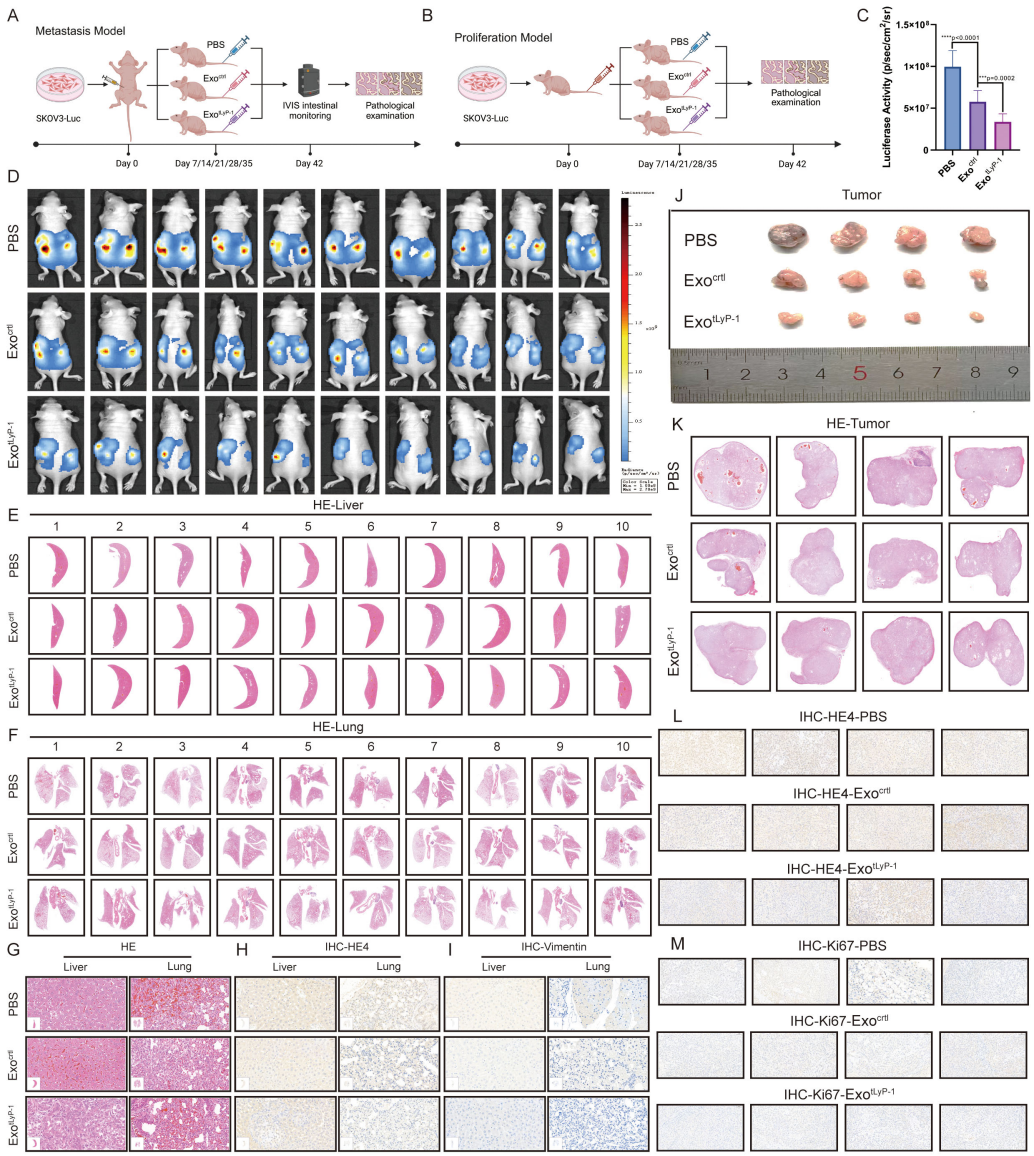
*In vivo* anti-tumor effect of exo^tLyP-1^. **(A)** Schematic of the mouse abdominal hormone-dependent tumor model. Exosomes (100 μg) were administered every 7 days (five total injections). PBS served as the control. **(B)** Schematic of the mouse subcutaneous hormone-dependent tumor model. Exosomes (100 μg) were administered every 7 days (five total injections). PBS served as the control. **(C, D)***In vivo* fluorescence imaging of metastases at day 42 (n = 10). Radiance scale: min = 1 × 10^8^, max = 2.78 × 10^9^ (p/s/cm²/sr). **(E-G)** HE staining of lung and liver metastases in exo^tLyP-1^, exo^ctrl^, and PBS groups (n = 10). Scale bar= 500 μm **(E, F)**; 20 μm **(G)**. **(H, I)** Immunohistochemical analysis of HE4 and vimentin expression in liver **(H)** and lung **(I)** metastatic nodules (n = 10). Scale bar = 20 μm. **(J)** Representative xenograft tumors per group (n = 4). **(K)** Tumor histopathology assessed by HE staining (n = 4). Scale bar = 500 μm. **(L, M)** Immunohistochemical analysis of HE4 **(L)** and Ki67 **(M)** expression in tumor tissue (n = 4). Scale bar = 20 μm. Data are shown as mean ± standard deviation values; ns *P* ≥ 0.05, ***P < 0.001, ****P < 0.0001.

To investigate the potential anti-tumor efficacy of NK92 cell-derived exosomes *in vivo*, SKOV3 cells were transplanted into mice via intraperitoneal or subcutaneous injection. Mice were randomized into groups on postoperative day 7 and subsequently treated via tail vein injection every 7 days for a total of five administrations (**Figures 4A, B**).

In the intraperitoneal tumor-bearing model, tumor progression was monitored by bioluminescence imaging on postoperative day 42. PBS-treated mice exhibited rapid tumor growth and intense bioluminescence. In contrast, significantly reduced bioluminescence intensity was observed in both the exo^ctrl^ and exo^tLyP-1^-treated groups compared to the PBS group, indicating a therapeutic response against ovarian cancer in the former (**Figures 4C, D**). Notably, the exo^tLyP-1^ group displayed a significantly smaller bioluminescent area and weaker intensity than the exo^ctrl^ group, suggesting exo^tLyP-1^ confers superior anti-tumor activity.

Given the propensity of ovarian cancer cells to metastasize to the liver and lungs, we assessed the effects of exo^tLyP-1^ on metastatic potential through pathological analysis of these organs. HE staining revealed metastatic lesions characteristic of ovarian cancer in mouse livers and lungs (**Figures 4E–G**). Subsequent detection of the ovarian cancer-specific marker HE4 in these tissues corroborated the staining findings (**Figure 4H**; **Supplementary S2A, B**). Furthermore, expression of the mesenchymal marker vimentin, indicative of migratory potential, was significantly lower in the exo^tLyP-1^ group compared to both the PBS and exo^ctrl^ groups (**Figure 4I**; **Supplementary S2C, D**). This reduction confirms that exo^tLyP-1^ inhibits ovarian cancer cell migration.

In the subcutaneous tumor model, tumor volume was significantly reduced in both the exo^ctrl^- and exo^tLyP-1^-treated groups relative to the PBS group, demonstrating inhibition of malignant proliferation (**Figure 4J**). Histopathological analysis of tumor tissues via HE staining confirmed characteristic ovarian cancer malignancy (**Figure 4K**). Immunohistochemical analysis of the ovarian cancer marker HE4 and the proliferation marker Ki67 further demonstrated that both exo^ctrl^ and exo^tLyP-1^ inhibit ovarian cancer cell proliferation, with the inhibitory effect of exo^tLyP-1^ being more pronounced (**Figures 4L, M**; **Supplementary S2E, F**).

### 3.5 NK92 cell-derived exosomes act via miR-31-5p-*GPRC5A* axis to inhibit ovarian cancer progression

**Figure 5:**
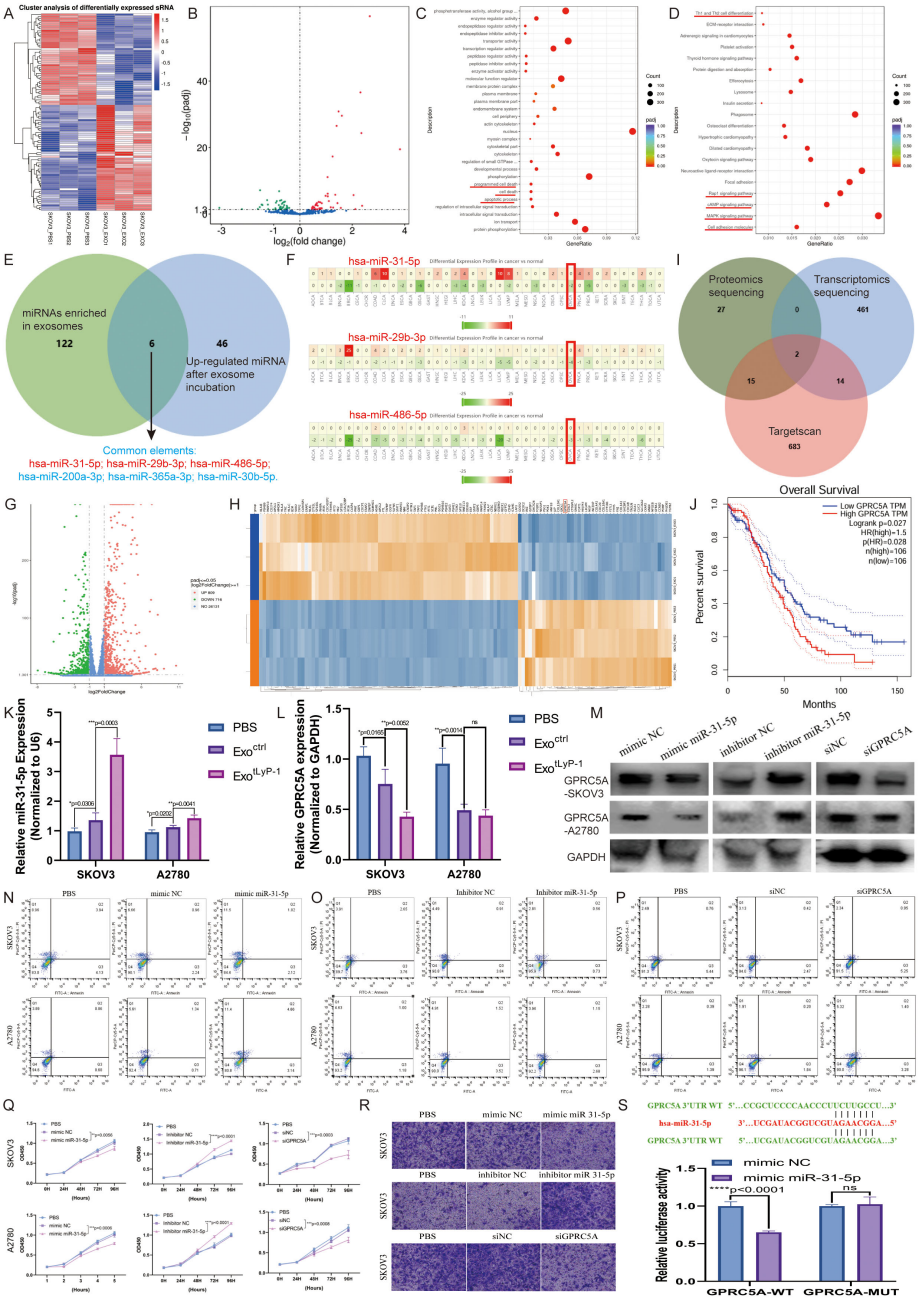
NK92 cell-derived exosomes via miR-31-5p-*GPRC5A* axis to inhibit ovarian cancer progression. **(A)** Unsupervised clustering of differentially expressed small RNAs (n = 3). Red and blue denote high and low miRNA expression, respectively. **(B)** Volcano plot of differentially expressed miRNAs. The x-axis represents log_2_-transformed fold change (FC); the y-axis indicates the statistical significance (-log_1_\_0_[*P* value]). Thresholds: |log_2_FC| ≥ 2, *P* < 0.05. **(C)** GO analysis of 52 upregulated miRNAs. The x-axis shows the enrichment ratio; the y-axis lists GO terms. **(D)** KEGG pathway analysis of 52 upregulated miRNAs. The x-axis represents the Rich factor; the y-axis lists pathway names. **(E)** Venn diagram identifying miRNAs enriched in NK92 cell-derived exosomes overlapping with miRNAs upregulated in SKOV3 cells post co-incubation. **(F)** Meta-analysis of miRNA (has-miR-31-5p, has-miR-29b-3p, and has-miR-486-5p) expression profiles across cancers using the dbDEMC database. **(G)** Volcano plot of differentially expressed mRNAs following co-incubation with NK92-derived exosomes. Thresholds: |log_2_FC| ≥ 1, *P* < 0.05. **(H)** Heatmap of differentially expressed mRNAs (n = 3) post co-incubation. **(I)** Venn diagram intersecting TargetScan-predicted miRNA targets with mRNAs and proteins downregulated in SKOV3 post co-incubation (*GPRC5A* and *SPARC*). **(J)** Correlation between *GPRC5A* expression and overall survival (obtained from GEPIA database). **(K)** miR-31-5p levels in ovarian cancer cell lines treated with exo^ctrl^ (30 μg) or exo^tLyP-1^ (30 μg), relative to PBS controls. **(L)***GPRC5A* protein levels in ovarian cancer cell lines treated with exo^ctrl^ (30 μg) or exo^tLyP-1^ (30 μg), relative to PBS controls. **(M)** Western blot analysis of *GPRC5A* expression in SKOV3 and A2780 cells transfected with miR-31-5p mimics, inhibitors, or si*GPRC5A*. **(N-P)** Apoptosis rates in SKOV3 and A2780 cells transfected miR-31-5p mimics, inhibitors, or si*GPRC5A*, assessed by flow cytometry. The total number of cells in regions Q2 and Q3 in the figure represents the total number of apoptotic cells. **(Q)** Proliferation efficiency of transfected cells with miR-31-5p mimics, inhibitors, or si*GPRC5A* measured by CCK-8 assay (n = 4). **(R)** Migration efficiency of transfected SKOV3 cells with miR-31-5p mimics, inhibitors, or si*GPRC5A* evaluated via Transwell assay. Scale bar = 100 μm. Representative images are shown. **(S)** Relative luciferase activities were measured in 293T cells transfected with either *GPRC5A*-wt or *GPRC5A*-mut plasmids alongside miR-31-5p mimics or negative control mimics. Data are shown as mean ± standard deviation values; ns *P* ≥ 0.05, **P < 0.01, ***P < 0.001, ****P < 0.0001.

To investigate the anti-tumor mechanism of NK92 cell-derived exosomes in ovarian cancer, we performed small RNA-seq on SKOV3 cells following co-incubation with exosomes (**Figure 5A**). Compared to using the PBS control, incubation with NK92 cell-derived exosomes upregulated 52 miRNAs (**Figure 5B**). GO and KEGG analyses revealed that these miRNAs modulate multiple signaling pathways critical for cell survival and metastasis (**Figures 5C, D**). Intersection of miRNAs enriched in NK92 cell-derived exosomes with those upregulated in SKOV3 cells identified the following six candidates: hsa-miR-31-5p, hsa-miR-200a-3p, hsa-miR-29b-3p, hsa-miR-365a-3p, hsa-miR-30b-5p, and hsa-miR-486-5p (**Figure 5E**). Cross-referencing with the dbDEMC cancer miRNA database confirmed three of these to be suppressors of ovarian cancer, with miR-31-5p exhibiting the greatest abundance (**Figure 5F**).

As miRNAs typically function through mRNA degradation, we conducted transcriptomic and proteomic sequencing of exosome-treated SKOV3 cells to identify miR-31-5p targets (**Figures 5G, H**). Integration of TargetScan predictions with downregulated transcripts and proteins identified two candidates: *GPRC5A* and *SPARC* (**Figure 5I**). Notably, high *GPRC5A* expression correlated with poor overall survival in ovarian cancer patients, while *SPARC* expression showed no significant association, implicating *GPRC5A* in disease progression (**Figure 5J**, **S3A****Supplementary S3**).

To validate targeting, we quantified miR-31-5p and *GPRC5A* expression in SKOV3 cells incubated with exo^ctrl^ or exo^tLyP-1^. Exosomal incubation upregulated miR-31-5p and downregulated *GPRC5A* (**Figures 5K, L**), suggesting a regulatory relationship. Protein-level validation using miR-31-5p mimics/inhibitors and *GPRC5A* siRNA confirmed inverse correlation, while miR-31-5p overexpression did not alter *SPARC* (**Figure 5M**, **Supplementary S3B**). Dual-luciferase reporter assays in 293T cells demonstrated direct binding of miR-31-5p to the *GPRC5A* 3′UTR (**Figure 5S**).

Functional assays revealed that miR-31-5p promotes apoptosis and suppresses proliferation/metastasis in ovarian cancer cells by degrading *GPRC5A* (**Figures 5N–R**). Consistent with this, analysis of *in vivo* mouse models (subcutaneous/intraperitoneal) showed that exosome treatment reduced *GPRC5A* expression, inhibiting tumor growth and metastasis (**Supplementary Figure S3C**).

Rescue experiments using miR-31-5p inhibitor and *GPRC5A* siRNA confirmed that *GPRC5A* knockdown reverses the pro-tumorigenic effects of miR-31-5p suppression (**Supplementary Figures S3D–G**). Also, quantitative PCR indicated functional delivery of 5%-15% of exosomal miR-31-5p to recipient cells (**Supplementary Figure S4A**). Collectively, these data demonstrate that *GPRC5A* is a direct target of miR-31-5p and mediates its anti-tumor effects.

### 3.6 NK92 cell-derived exosomes enhance DDP sensitivity of ovarian cancer cells by targeted delivery of ABCB1 siRNA

**Figure 6:**
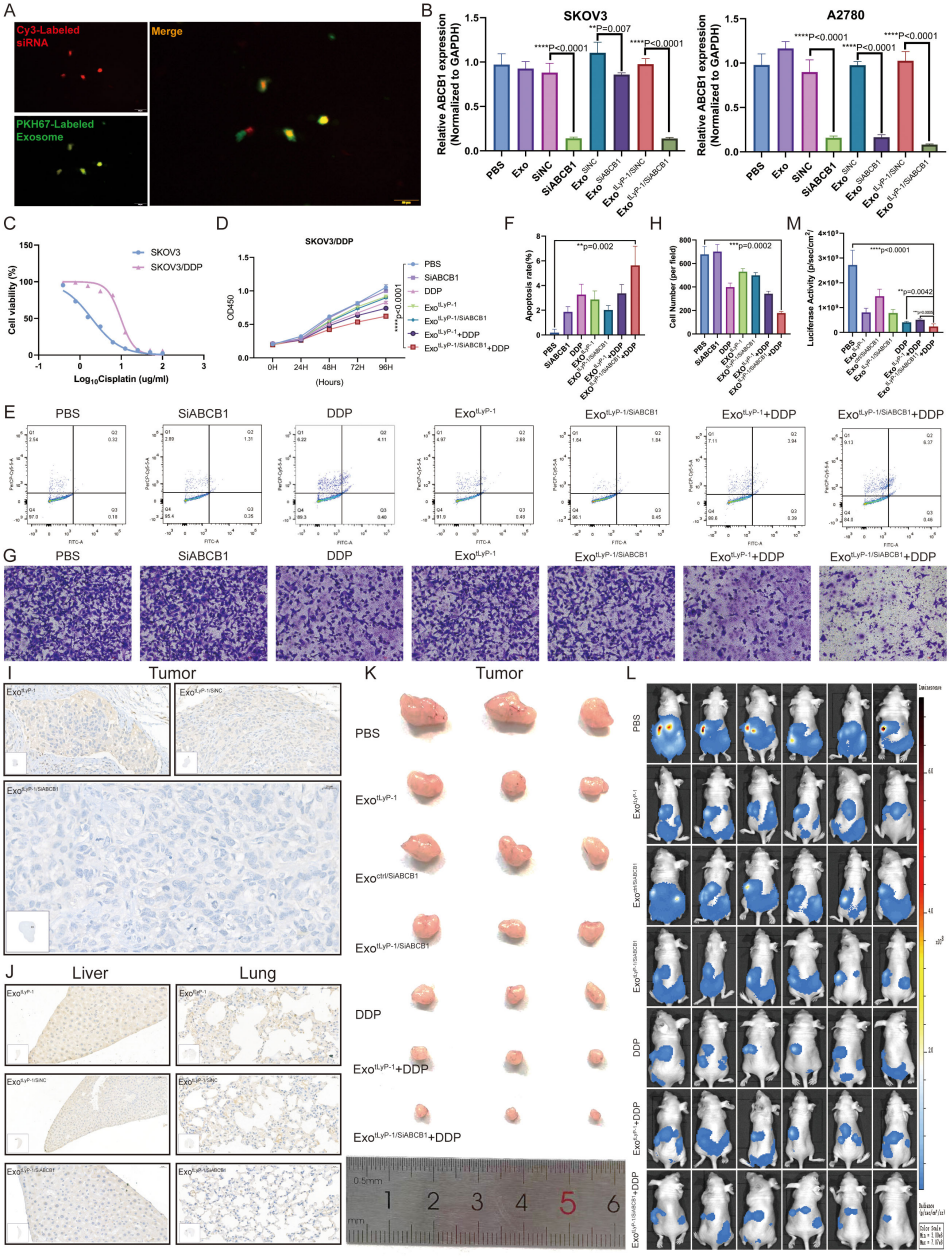
Combination of exo^tLyP-1/SiABCB1^ and DDP inhibits progression of SKOV3/DDP. **(A)** Confocal microscopy and co-localization analysis of Cy3-labeled siRNA (red) and PKH67-labeled exosomes (green). Scale bar = 10 µm. **(B)***ABCB1* expression levels in SKOV3 and A2780 cells under different treatment conditions. **(C)** Half-maximal inhibitory concentration (IC_50_) of cisplatin in SKOV3/DDP cells. **(D-H)** Cell proliferation (CCK-8 assay), apoptosis rates (flow cytometry), and migration capacity (Transwell assay) of SKOV3/DDP cells under different treatments. The total number of cells in regions Q2 and Q3 in the figure represents the total number of apoptotic cells. **(I, J)** Immunohistochemical analysis of ABCB1 expression in tumor, liver, and lung tissues (n = 3). Scale bar = 20 µm. **(K)** Representative images of xenograft tumors under different treatment conditions (n = 3). **(L, M)***In vivo* fluorescence imaging of metastatic burden under various conditions at day 42 (n = 6; Radiance scale: min = 3 × 10^6^, max = 7.07 × 10^8^, p/sec/cm^2^/sr). Data are shown as mean ± standard deviation values; ns *P* ≥ 0.05, **P < 0.01, ***P < 0.001, ****P < 0.0001.

To preserve the integrity of NK92 cell-derived exosomes and facilitate ABCB1 siRNA delivery into recipient cells, we employed the cholesterol-modification strategy established by Kang et al., wherein siRNA adsorption to exosomal membranes is enabled via cholesterol conjugation. Cholesterol-modified ABCB1 siRNA was synthesized and co-incubated with exosomes. Laser confocal microscopy revealed co-localization of CY3-labeled ABCB1 cholesterol-modified siRNAs and PKH67-labeled exosomes, confirming successful siRNA loading onto exosomes (**Figure 6A**). Fluorescence intensity quantification indicated ~70% loading efficiency. Furthermore, ~40% of the loaded siRNA was internalized and functionally delivered to recipient cells (**Supplementary Figures S4B, C**).

Following 24-h co-incubation of ovarian cancer cells with exosomes (exo^ctrl^ or exo^tLyP-1^) loaded with cholesterol-modified ABCB1 siRNA, qRT-PCR detected significant gene silencing (**Figure 6B**). To assess whether exo^tLyP-1/SiABCB1^ sensitizes chemoresistant ovarian cancer cells to therapeutics, we generated a SKOV3/DDP cell line (**Figure 6C**). Combining exo^tLyP-1/SiABCB1^ with DDP significantly inhibited SKOV3/DDP proliferation (CCK-8 assay; **Figure 6D**), enhanced apoptosis (flow cytometry; **Figures 6E, F**), and reduced migration (Transwell assay; **Figures 6G, H**) compared to controls.

For *in vivo* evaluation, mice bearing subcutaneous or peritoneal xenografts received tail vein injections of exo^tLyP-1/SiABCB1^. ABCB1 expression was markedly reduced in tumors, livers, and lungs (**Figures 6I, J**; **Supplementary S4D, E**), demonstrating functional siRNA delivery via exosomes. Moreover, exo^tLyP-1/SiABCB1^ combined with DDP robustly suppressed tumor growth and metastasis *in vivo* relative to PBS or monotherapy (**Figures 6K–M**). These results establish that NK92 cell-derived exosome-mediated siABCB1 delivery synergizes with DDP to inhibit ovarian cancer progression, revealing a promising translational strategy.

## 4 Discussion

Nanoparticles have been extensively studied as drug-delivery vehicles and have shown excellent results in the treatment of disease over the past decades (20, 21). In cancer therapy, they offer exciting solutions to improve efficacy by prolonging the residence time of the drug inside the tumor tissue while minimizing the accumulation of the drug in normal tissues to overcome side effects (22). As one example, the albumin-bound nanoparticle (nab) paclitaxel, which promotes drug tissue distribution and tumor penetration, has become a highly successful anti-tumor nanomedicine for clinical applications following the study by *Chen* et al. (23). Nonetheless, most nanomedicines carry their own toxic components (causing immunotoxicity, inflammation, etc.) that still significantly limit their translational applications, and therefore new carriers are urgently needed (24). As a new type of carrier, exosomes are safer to use, can be similarly employed for targeted delivery of drugs, and have demonstrated their therapeutic potential in preclinical studies. For example, the use of doxorubicin-loaded exosomes has been shown to be more effective in inhibiting tumor growth with fewer side effects than the equivalent free drug (25). Second, exosome-based engineered modification strategies can overcome tumor cell resistance to therapeutic drugs by altering the drug efflux system. It has been demonstrated that the use of exosomes as drug-delivery vehicles in the treatment of drug-resistant cells can show more potent drug cytotoxicity and anti-tumor effects (26). Notably, drugs delivered by exosomes exert their effects only if they accurately enter the target cells (27). Therefore, to further improve the targeting of exosomes, two main strategies are currently used (28). The first approach involves exogenous modification of the exosome surface to produce actively targeted exosomes. For example, Li et al. demonstrated that A33-positive exosomes can form complexes with A33 antibody-coated superparamagnetic iron oxide nanoparticles (SPION) to obtain targeting of A33-positive colon cancer cells (29). The second approach is the genetic modification of the exosome surface to display the targeting peptide or antibody. In Alzheimer’s disease studies, RVG peptides expressed together with the exosome membrane surface protein Lamp2b effectively introduced targeted delivery of BACE1 siRNA into the mouse brain, with gene-silencing effects (30).

Despite demonstrating significant clinical potential as emerging cell-free therapeutics across multiple disease areas, exosomes still face multifaceted challenges to their large-scale clinical translation, including those related to standardization of manufacturing, long-term safety, and targeted delivery. First, the yield of engineered exosomes is influenced by cell sources, culture sera, and isolation methods, with variations in culture conditions and extraction protocols, compromising batch-to-batch homogeneity and subpopulation consistency; thus, scalable cell cultivation and standardized isolation workflows remain critical unmet needs. Second, while rigorous *in vitro* characterization and quantification are essential for predicting therapeutic efficacy of drug-loaded exosomes, establishing universal “gold standards” for exosome quantification, molecular profiling, and physical characterization requires further work. Moreover, industrial-scale production of high-purity, homogeneous engineered exosomes presents substantial technical barriers, particularly due to difficulties in isolating exosomes from complex biological matrices. Conventional laboratory-scale ultracentrifugation—when scaled up—suffers from high energy consumption, low recovery rates (typically <30%), and vesicle membrane damage that compromises functionality. Critically, uncertainties persist regarding long-term safety, biodistribution, and immune responses to repeated administrations. No standardized quality-control protocols or regulatory guidelines currently exist for engineered exosomes. Long-term risks remain undefined due to exosomes’ capacity for intercellular communication and recipient cell modulation. Biodistribution pathways in humans—including absorption, organ-specific accumulation, and excretion—require longitudinal tracking to assess off-target toxicity, and targeted delivery efficiency to specific tissues/cells demands deeper investigation. Repeated injections may provoke immune responses despite low inherent immunogenicity; however, clinical data on immunogenicity and its impact on therapeutic efficacy remain insufficient. Consequently, while exosome-based therapies hold considerable promise, overcoming these technical and safety hurdles is imperative for scalable clinical implementation.

In this study, we engineered NK92 cells to express a Lamp2b-tLyP-1 fusion protein for the generation of engineered exosomes targeting ovarian cancer cells. Among them, the membrane-penetrating peptide tLyP-1 has been shown to specifically target binding to NRP-1 proteins that are highly expressed in tumors and also to penetrate tumor vasculature and stroma to reach deep into the tumor (31). It has been shown that NK92 cells are able to exert tumor-killing functions through the perforin/granzyme pathway, the death ligand pathway, and the cytokine pathway (32, 33). Compared to previous studies, we analyzed the composition of NK92 cells for the first time in this study and demonstrated that NK92 cell-derived exosomes containing miR-31-5p affect ovarian cancer progression by inhibiting *GPRC5A* expression.

Most ovarian cancer patients are initially sensitive to chemotherapy with platinum-based agents, but primary platinum resistance usually develops with time on treatment, which in turn constitutes a major challenge in clinical management (33, 34). Among multiple molecular mechanisms, platinum resistance is usually due to excessive drug efflux and is mediated by the ATP-binding cassette (ABC) transporter (35). It has been shown that the expression of ABC transporter proteins, particularly multidrug resistance protein 1 (MDR1), is closely associated with tumor development, and that many chemotherapeutic drugs, such as uptake and distribution, are affected by ABCB1. MDR1, encoded by member B of ABCB1, confers resistance to drug toxicity and chemotherapy and is the target of many anticancer drugs. Overexpression of ABCB1 often leads to multidrug resistance in cancer cells (36). A recent meta-analysis of 8607 patients confirmed that overexpression of ABCB1 is associated with chemotherapy resistance and poor prognosis in ovarian cancer patients (37). Interestingly, our findings suggest that engineered exosomes are more efficient at delivering ABCB1 siRNA into tumor cells to function and, in combination with DDP, can treat drug-resistant ovarian cancer.

## 5 Conclusion

**Figure 7:**
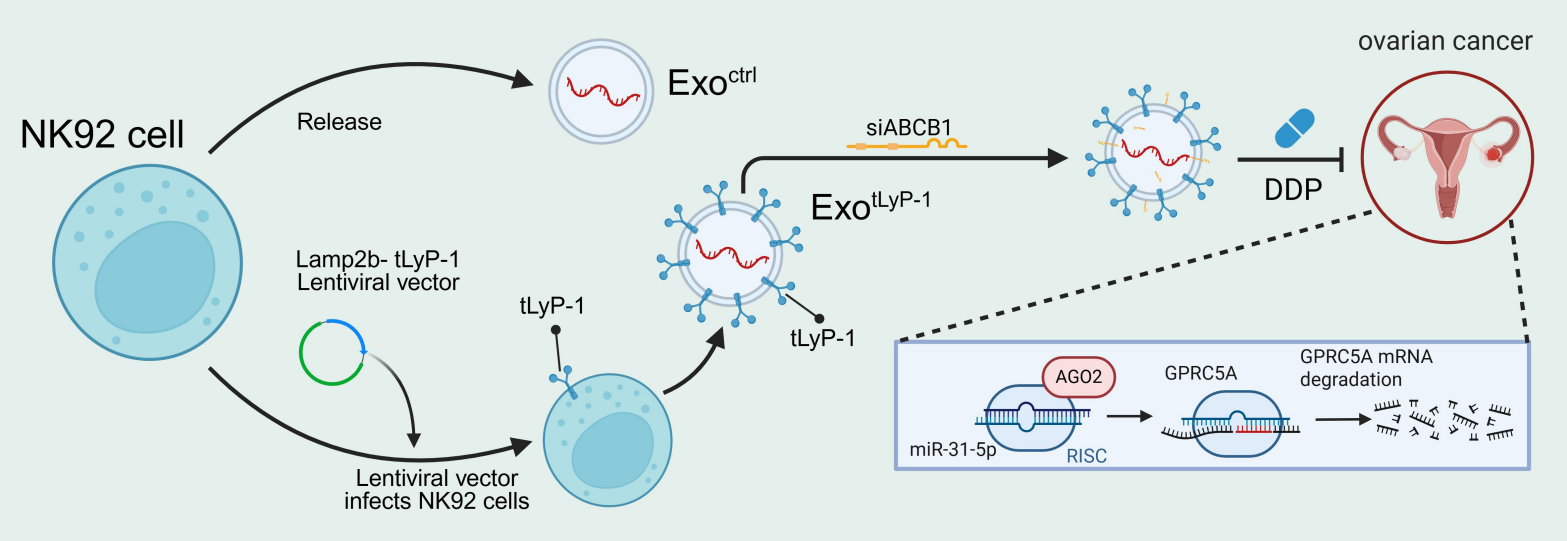
Engineered NK92 cell-derived exosomes inhibit ovarian cancer progression by degrading *GPRC5A*.

In this study, we characterized the protein and miRNA profiles of NK92 cells and their exosomes, establishing their capacity to inhibit ovarian cancer progression through GPRC5A degradation. Concurrently, we developed a genetic-engineering method to produce modified exosomes by fusing the cell-penetrating peptide tLyP-1 to Lamp2b. Furthermore, exosomes displaying tLyP-1 (exo^tLyP-1^) effectively delivered ABCB1 siRNA into ovarian cancer cells, enhancing tumor cell drug sensitivity (**Figure 7**). Collectively, our findings indicate that combining DDP with an NK92-derived exosomal siRNA delivery system represents a promising therapeutic strategy for drug-resistant ovarian cancer.

## Data Availability

The data presented in the study are deposited in the Genome Sequence Archive (Genomics, Proteomics & Bioinformatics 2025) in National Genomics Data Center (Nucleic Acids Res 2025), China National Center for Bioinformation / Beijing Institute of Genomics, accession number OMIX012617: https://ngdc.cncb.ac.cn/omix/release/OMIX012617, OMIX012648: https://ngdc.cncb.ac.cn/omix/release/OMIX012648, HRA014310: https://ngdc.cncb.ac.cn/gsa-human/browse/HRA014310, HRA014312: https://ngdc.cncb.ac.cn/gsa-human/browse/HRA014312, HRA014322: https://ngdc.cncb.ac.cn/gsa-human/browse/HRA014322.

## Supplementary material

Supplementary Figure 1 **(A)** The dbDEMC database was used to count the expression of miRNAs enriched in NK92-derived exosomes in different cancer species.

Supplementary Figure 2 **(A, B)** IHC analysis of HE4 expression in liver and lung tissues (n = 10). Scale bar = 500 µm. **(C, D)** IHC analysis of vimentin expression in liver and lung tissues (n = 10). Scale bar = 500 µm. **(E, F)** IHC analysis of HE4 and Ki67 expression in tumor tissues (n = 4). Scale bar = 20 µm.

Supplementary Figure 3 **(A)** Correlation between *SPARC* expression and overall survival (data sourced from the GEPIA database). **(B)** Expression of *SPARC* in SKOV3 and A2780 cells remained unchanged following treatment with miR-31-5p mimics. **(C)** Immunohistochemical detection of *GPRC5A* in tumors (n = 4), lungs (n = 10), and livers (n = 10) from mice treated with exo^ctrl^ or exo^tLyP-1^.**(D)** Cell proliferation was assessed by CCK-8 assay after transfection with miR-31-5p inhibitor and si*GPRC5A*. **(E, F)** Migration efficiency of SKOV3 and A2780 cells treated with miR-31-5p inhibitor and si*GPRC5A* was evaluated using Transwell assays (n = 3). Scale bar = 100 μm. **(G)** Apoptosis rates in SKOV3 and A2780 cells treated with miR-31-5p inhibitor and si*GPRC5A* were determined by flow cytometry. Data are shown as mean ± standard deviation values; ns *P* ≥ 0.05, **P* < 0.05, ***P* < 0.01, ****P* < 0.001, *****P* < 0.0001.

Supplementary Figure 4 **(A)** miR-31-5p expression levels in NK92 cell-derived exosomes (30 μg), SKOV3 cells, and SKOV3 cells incubated with NK92 cell-derived exosomes (30 μg). **(B)** NK92 cell-derived exosomes (30 μg) were mixed with cholesterol-modified ABCB1 siRNA (100 nM) in 100 μL of PBS and incubated at 37 °C for 1 h. Residual ABCB1 siRNA in the mixture was quantified. After mixing, samples were concentrated and centrifuged (washed), and the pellet was resuspended in PBS. Total ABCB1 siRNA in the pellet was determined by comparing fluorescence signals of exosome-containing samples (+Exo) and exosome-free controls (−Exo) to a standard curve of input siRNA (100%). **(C)** Exo^SiABCB1^ (30 μg, exosomes loaded with cholesterol-modified ABCB1 siRNA) was mixed with 1 × 10^5^ SKOV3 cells in 200 μL of PBS at 37 °C for 1 h. Residual exo^SiABCB1^ was quantified. Following co-incubation, samples were centrifuged (washed), and the pellet was resuspended in PBS. Total ABCB1 siRNA in the pellet was evaluated by comparing fluorescence signals of cell-containing samples (+SKOV3) and cell-free controls (−SKOV3) to a standard curve of input exo^SiABCB1^ (100%). **(D, E)** Immunohistochemical analysis of ABCB1 expression in liver, lung, and tumor tissues (n = 4). Scale bar = 500 μm. Data are shown as mean ± standard deviation values; ns *P* ≥ 0.05, **P* < 0.05, ***P* < 0.01, ****P* < 0.001, *****P* < 0.0001.
