# Supplementary figures and images for: Engineered NK92 cell-derived exosomes inhibit ovarian cancer progression by degrading GPRC5A
Source: Front Immunol. 2025 Nov 11;16:1613178. doi: 10.3389/fimmu.2025.1613178 (PMC12643886; doi:10.3389/fimmu.2025.1613178)

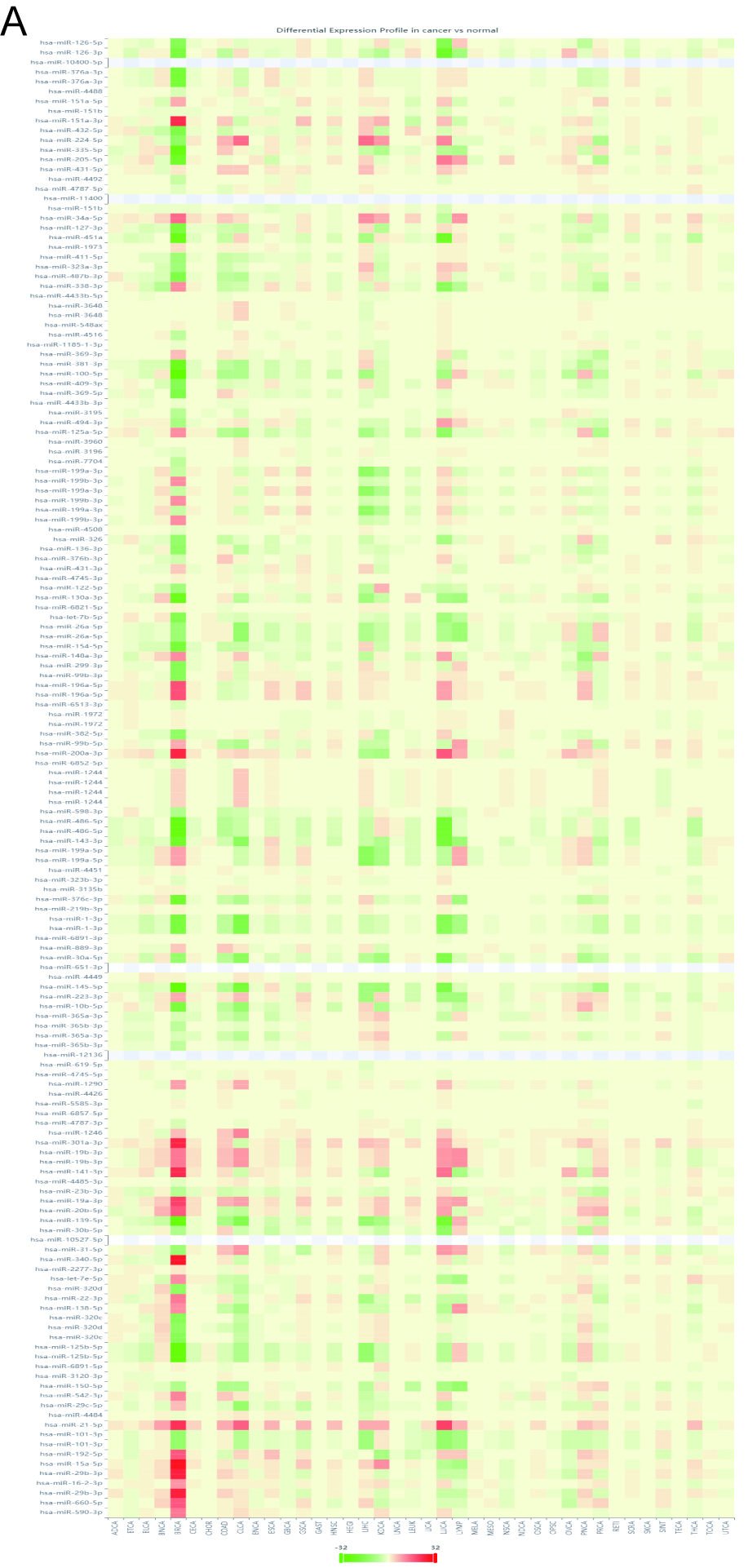

Supplement: Supplementary Figure 1 — (A) The dbDEMC database was used to count the expression of miRNAs enriched in NK92-derived exosomes in different cancer species. [file Image1.jpeg]

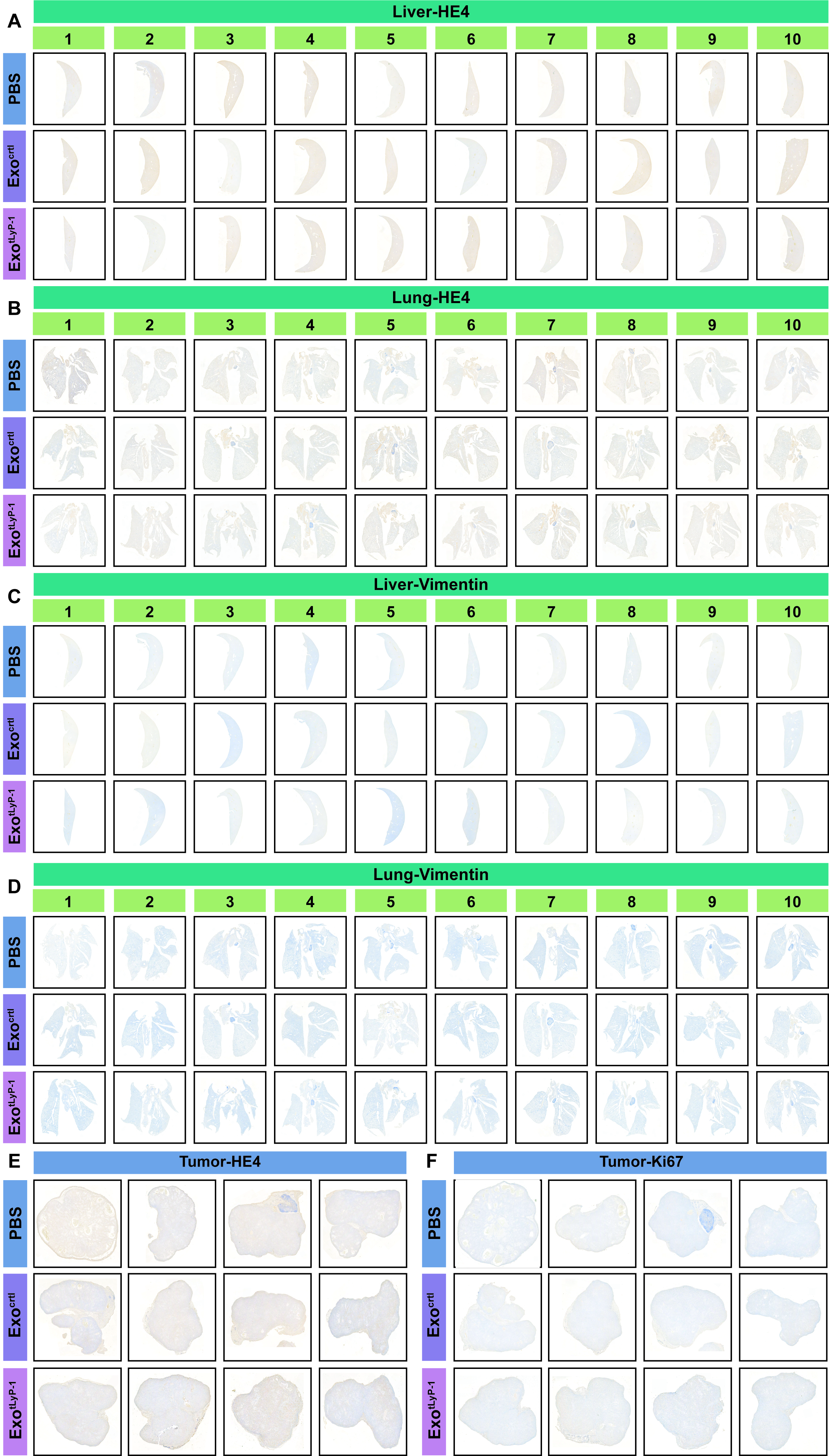

Supplement: Supplementary Figure 2 — (A, B) IHC analysis of HE4 expression in liver and lung tissues (n = 10). Scale bar = 500 µm. (C, D) IHC analysis of vimentin expression in liver and lung tissues (n = 10). Scale bar = 500 µm. (E, F) IHC analysis of HE4 and Ki67 expression in tumor tissues (n = 4). Scale bar = 20 µm. [file Image2.jpeg]

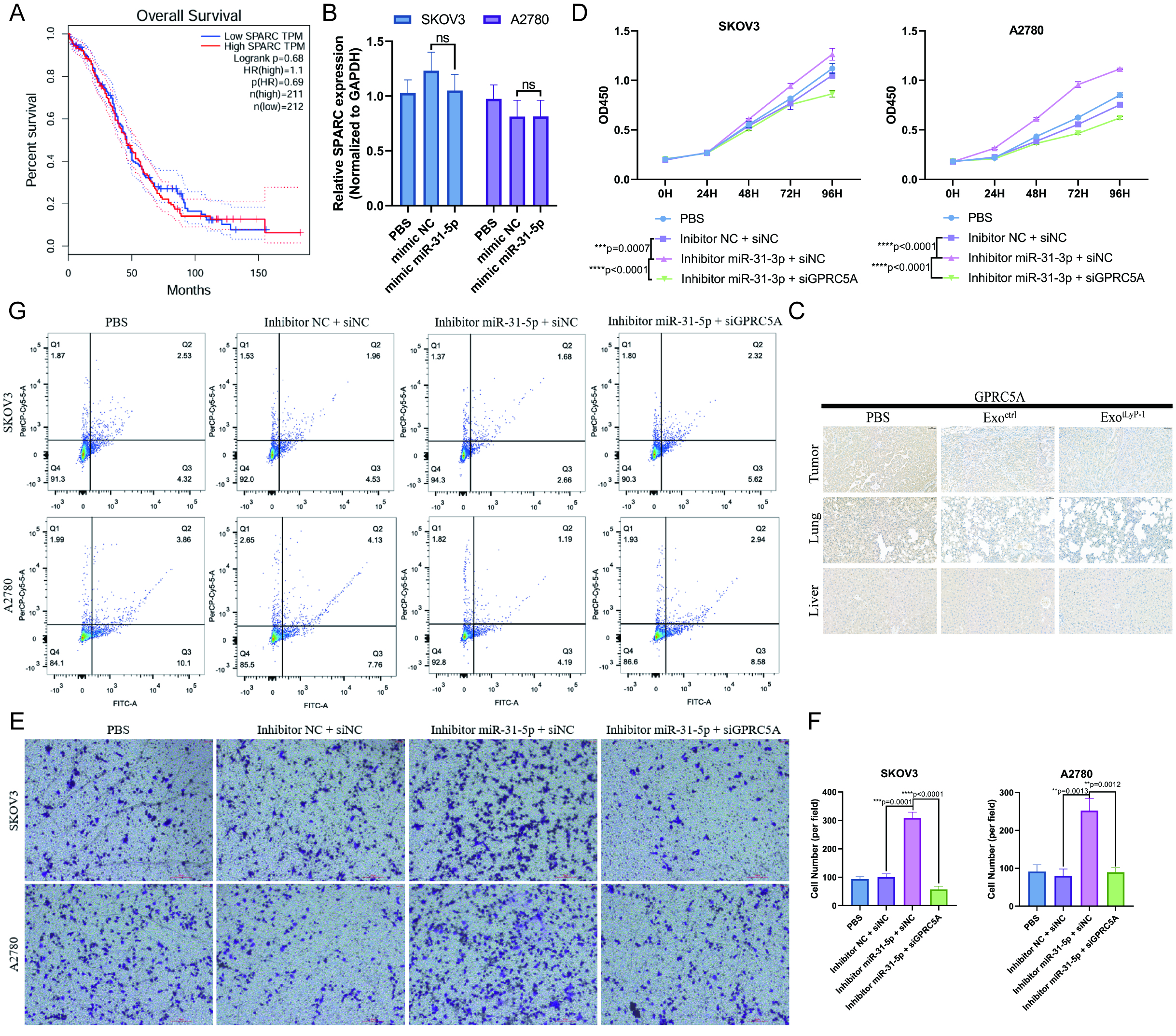

Supplement: Supplementary Figure 3 — (A) Correlation between SPARC expression and overall survival (data sourced from the GEPIA database). (B) Expression of SPARC in SKOV3 and A2780 cells remained unchanged following treatment with miR-31-5p mimics. (C) Immunohistochemical detection of GPRC5A in tumors (n = 4), lungs (n = 10), and livers (n = 10) from mice treated with exoctrl or exotLyP-1.(D) Cell proliferation was assessed by CCK-8 assay after transfection with miR-31-5p inhibitor and siGPRC5A. (E, F) Migration efficiency of SKOV3 and A2780 cells treated with miR-31-5p inhibitor and siGPRC5A was evaluated using Transwell assays (n = 3). Scale bar = 100 μm. (G) Apoptosis rates in SKOV3 and A2780 cells treated with miR-31-5p inhibitor and siGPRC5A were determined by flow cytometry. Data are shown as mean ± standard deviation values; ns P ≥ 0.05, *P < 0.05, **P < 0.01, ***P < 0.001, ****P < 0.0001. [file Image3.jpeg]

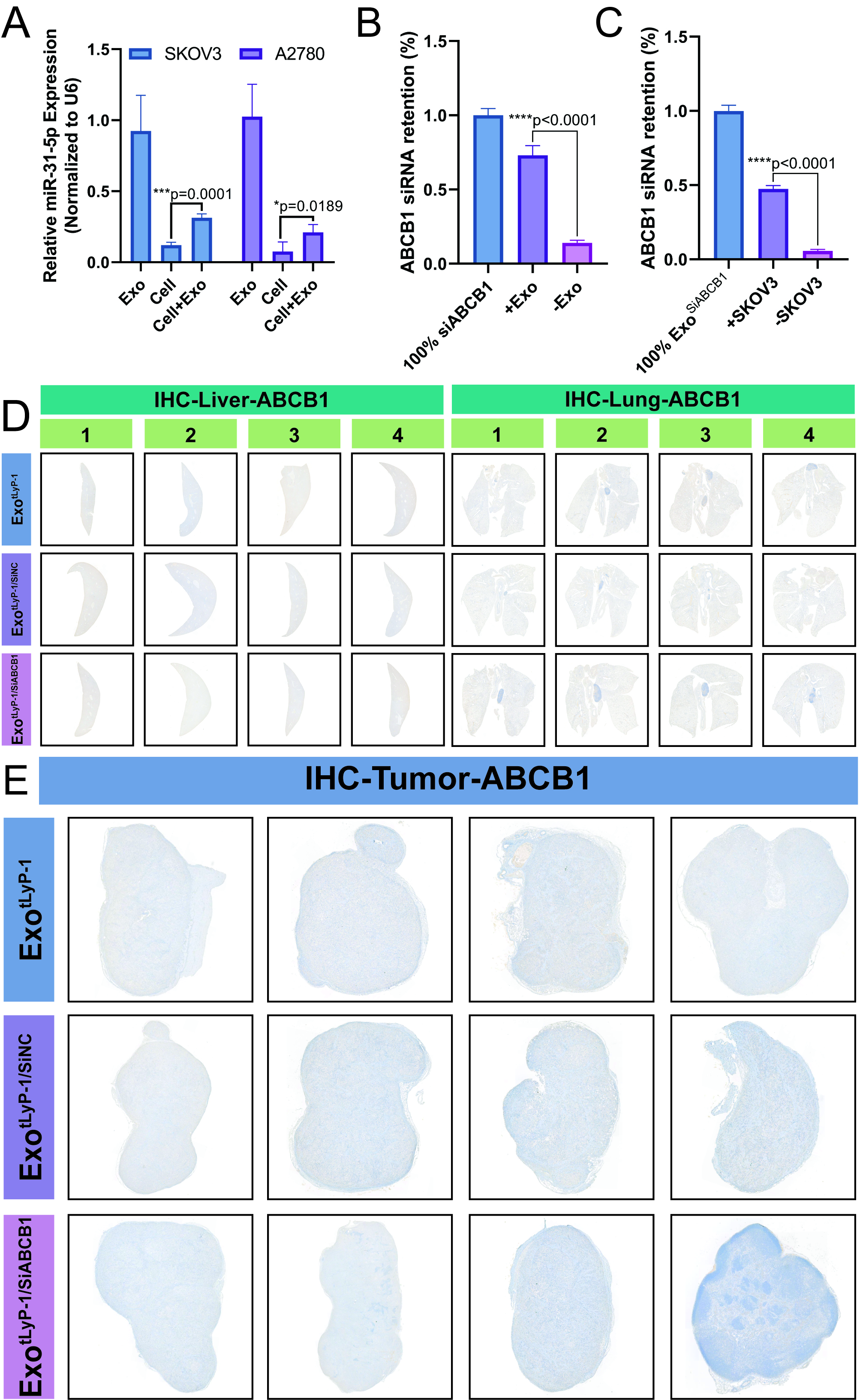

Supplement: Supplementary Figure 4 — (A) miR-31-5p expression levels in NK92 cell-derived exosomes (30 μg), SKOV3 cells, and SKOV3 cells incubated with NK92 cell-derived exosomes (30 μg). (B) NK92 cell-derived exosomes (30 μg) were mixed with cholesterol-modified ABCB1 siRNA (100 nM) in 100 μL of PBS and incubated at 37 °C for 1 h. Residual ABCB1 siRNA in the mixture was quantified. After mixing, samples were concentrated and centrifuged (washed), and the pellet was resuspended in PBS. Total ABCB1 siRNA in the pellet was determined by comparing fluorescence signals of exosome-containing samples (+Exo) and exosome-free controls (−Exo) to a standard curve of input siRNA (100%). (C) ExoSiABCB1 (30 μg, exosomes loaded with cholesterol-modified ABCB1 siRNA) was mixed with 1 × 10^5^ SKOV3 cells in 200 μL of PBS at 37 °C for 1 h. Residual exoSiABCB1 was quantified. Following co-incubation, samples were centrifuged (washed), and the pellet was resuspended in PBS. Total ABCB1 siRNA in the pellet was evaluated by comparing fluorescence signals of cell-containing samples (+SKOV3) and cell-free controls (−SKOV3) to a standard curve of input exoSiABCB1 (100%). (D, E) Immunohistochemical analysis of ABCB1 expression in liver, lung, and tumor tissues (n = 4). Scale bar = 500 μm. Data are shown as mean ± standard deviation values; ns P ≥ 0.05, *P < 0.05, **P < 0.01, ***P < 0.001, ****P < 0.0001. [file Image4.jpeg]
